# Supplementary material for: Effects of Personality and Behavioral Syndromes on Competition for Social Hierarchical Status in Anemonefish Amphiprion clarkii
Source: Animals (Basel). 2024 Jul 30;14(15):2216. doi: 10.3390/ani14152216 (PMC11311083; doi:10.3390/ani14152216)
Supplement: Supplementary file 1 [file animals-14-02216-s001.zip › animals-3083857-supplementary.pdf]

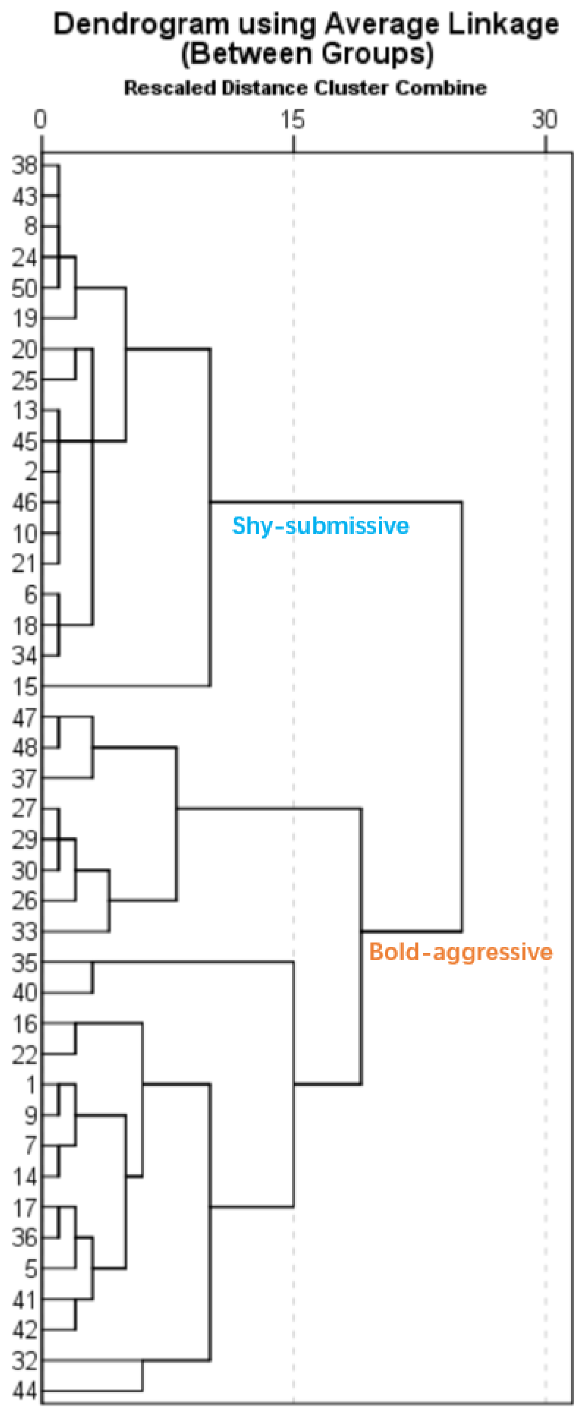

Figure S1. clustering plot showing individuals categorized into two personality types, bold-aggressive and shy-submissive.

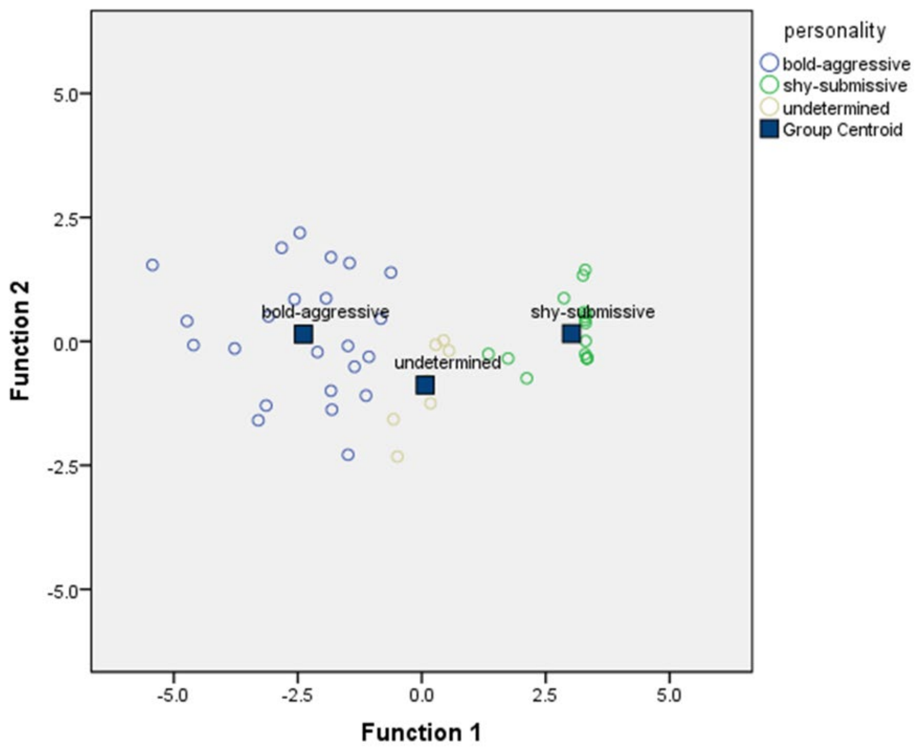

(a)

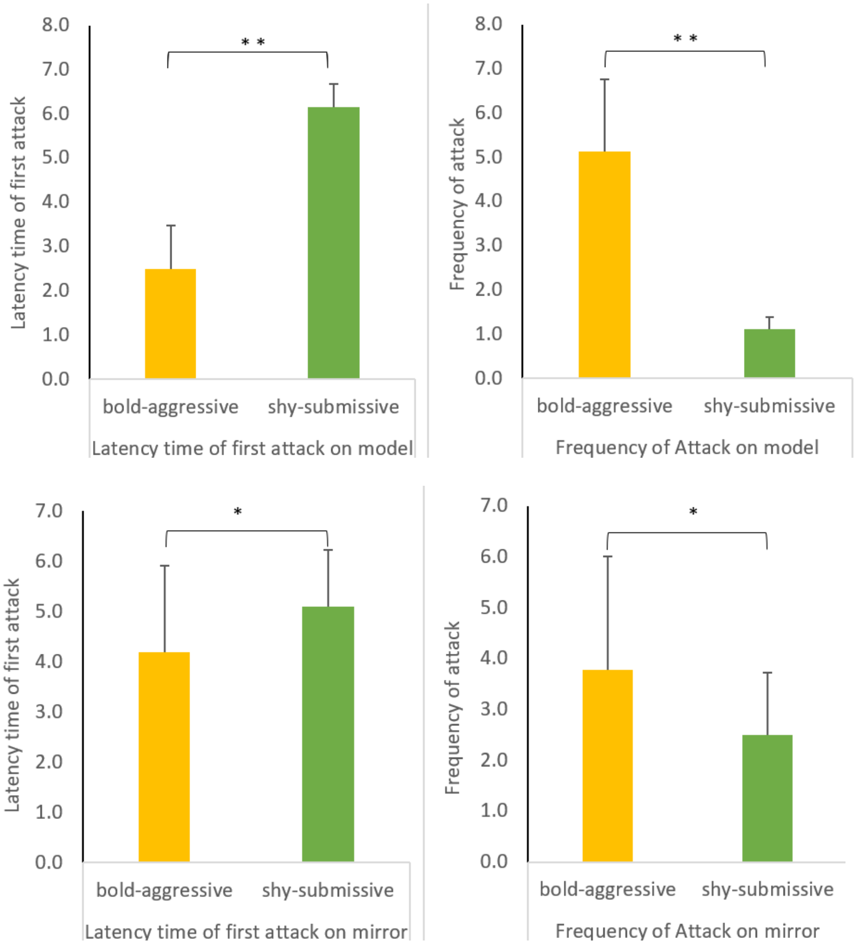

(b)

Figure S2. Differences between personality types. (a) Fisher's canonical discriminant analysis of individual personality type.(b) Means of the four behavioural variables in the personality assessment across the two personality types. Significant differences between bold-aggressive and shy-submissive types are indicated by an asterisk: * $p < 0.05$, ** $p < 0.01$.
